# Inflammation and Oxidative Stress in Seminal Plasma: Search for Biomarkers in Diagnostic Approach to Male Infertility

**DOI:** 10.3390/jpm12060857

**Published:** 2022-05-25

**Authors:** Carmine Bruno, Umberto Basile, Edoardo Vergani, Cecilia Napodano, Alessandro Oliva, Francesca Gulli, Elisabetta Meucci, Andrea Silvestrini, Patrick Orlando, Sonia Silvestri, Luca Tiano, Antonio Mancini

**Affiliations:** 1 Dipartimento di Medicina e Chirurgia Traslazionale, Università Cattolica del S. Cuore, 00168 Rome, Italy; carmine.bruno@outlook.it (C.B.); edoardo.vergani@outlook.it (E.V.); alessandro.oliva996@gmail.com (A.O.); 2 Fondazione Policlinico Universitario A. Gemelli IRCCS, 00168 Rome, Italy; umberto.basile@policlinicogemelli.it (U.B.); elisabetta.meucci@unicatt.it (E.M.); 3 Dipartimento di Scienze di Laboratorio e Infettivologiche, Fondazione Policlinico Universitario “A. Gemelli” IRCCS, Università Cattolica del Sacro Cuore, 00168 Rome, Italy; 4 Synlab s.r.l., 00168 Rome, Italy; cecilia.napodano@gmail.com; 5 Ospedale S. G. Vannini, 00168 Rome, Italy; dottfgulli@gmail.com; 6 Dipartimento di Scienze Mediche di base, Cliniche Anestesiologiche e Perioperatorie, Università Cattolica del S. Cuore, 00168 Rome, Italy; 7 Department of Life and Environmental Sciences, Polytechnical University of Marche, 60131 Ancona, Italy; p.orlado@univpm.it (P.O.); s.silvestri@univpm.it (S.S.); l.tiano@univpm.it (L.T.)

**Keywords:** free light chains, seminal fluid, varicocele, infertility, biomarkers

## Abstract

Oxidative and inflammatory damage underlie several conditions related to male infertility, including varicocele. Free light chains of immunoglobulins (FLCs) are considered markers of low-grade inflammation in numerous diseases. Coenzyme Q10 (CoQ10), a lipidic antioxidant and anti-inflammatory compound, is involved in spermatozoa energy metabolism and motility. We aimed to evaluate FLCs’ seminal levels in patients with varicocele in comparison to control subjects and to correlate them with CoQ10 and Total Antioxidant Capacity (TAC) in human semen. Sixty-five patients were enrolled. Semen analysis was performed; patients were divided into three groups: controls, 12 normozoospermic patients, aged 34 (33–41) years; varicocele (VAR), 29 patients, aged 33 (26–37) years; and idiopathic, 24 oligo-, astheno- and oligoasthenozoospermic patients aged 37 (33.5–40.5) years. FLCs (κ and λ) were assayed by turbidimetric method; CoQ10 by HPLC; TAC by spectrophotometric method. λ FLCs showed a trend toward higher levels in VAR vs. controls and the idiopathic group. VAR showed a trend toward lower κ FLCs levels vs. the other two groups. When comparing κ/λ ratio, VAR showed significantly lower levels vs. controls and idiopathic. Moreover, CoQ10 seminal levels showed higher levels in VAR and idiopathic compared to controls. Data reported here confirm lower levels of κ/λ ratio in VAR and suggest a possible application in personalized medicine as clinical biomarkers for male infertility.

## 1. Introduction

In the last few years, growing evidence has better elucidated the biological role of free light chains of immunoglobulin (FLCs), kappa (κ) and lambda (λ) [1,2]. As immunoglobulins (Ig) present a tetrameric structure, composed of two heavy chains and two light chains, for years, FLCs have been considered as a mere inconsequential spillover assembly, rather than bioactive molecules [1]. Nowadays, it is known that they may play enzymatic [3,4] and proteolytic [5,6] roles; they may activate complement cascade [7] and mast cells, with crucial role in the development of contact sensibility [8]; they may also inhibit the autonomous signaling ability of the B-cell receptor [9,10]. Plasmatic κ/λ ratio is considered a diagnostic parameter for multiple myeloma, as a ratio alteration could indicate a monoclonal FLCs production [11]. Polyclonal FLCs secretion has been correlated to inflammation [2]. Thus, the detection of FLCs in biological fluids may be useful as a biomarker of inflammation or altered immune response, as is reported above. In several diseases, a differential FLCs production pattern in plasma, urine, saliva, cerebrospinal and synovial fluid has been shown [12]. Our group have determined, for the first time, their detectability in seminal fluid with higher λ FLCs in semen characterized by inflammation and lower κ FLCs in seminal fluid of patients affected by varicocele (VAR) compared to control group [13].

VAR, the abnormal dilatation of the veins of pampiniform plexus, is one of the leading causes of male infertility, due to its relevant prevalence (about 15% of adult males) [14]. Male infertility is often related to oxidative damage inflicted on spermatozoa [15]. An altered oxidative balance with higher reactive oxygen species (ROS) production, higher lipid peroxidation and antioxidants responses, is well documented in VAR [16,17,18,19].

In oxidative balance, coenzyme Q10 (CoQ10) plays a crucial antioxidant role, with scavenging properties, to maintain cellular membrane integrity. In spermatozoa biology, it has a key role in energy metabolism, enabling mitochondrial oxidative phosphorylation and consequently supplying energy for their motility [20,21,22]. CoQ10 is detectable in human seminal fluid and shows direct correlation with seminal parameters [23]. Its antioxidant properties are directly related to sperm protection, as suggested by the inverse correlation between hydroperoxides and ubiquinol (CoQ10 reduced fraction) in seminal fluid [24]. Furthermore, CoQ10’s role as anti-inflammatory molecule has been recently evaluated. CoQ10 modulates gene expression [25], covering anti-inflammatory functions, even though a clear mechanism is not fully understood [26,27]. CoQ10 anti-inflammatory activity has not been addressed yet in infertile males. Moreover, due its lipophilic structure, CoQ10 can freely exchange from intra- and extra-cellular compartments. In this regard, VAR represents an interesting model, since we showed that VAR was an exception to the correlation described above between CoQ10 and seminal parameters [23]. In pre-operative VAR, higher plasmatic seminal levels have been described, even though only intracellular CoQ10 showed an inverse correlation with motility. After varicocelectomy, plasma-to-total CoQ10 ratio decreases and still no correlation between total seminal CoQ10 and motility is detected. As seminal plasma CoQ10 reflects an interchange between intra and extracellular compartments, its different distribution in VAR patients could define greater sensibility to peroxidative damage, reduced energy expenditure, and in turn defective motility [28,29].

The exact link between VAR and infertility is still an open issue. No correlation between seminal FLCs, oxidative stress parameters and CoQ10 biology has been investigated yet.

Thus, given the importance of oxidative stress in male infertility and the need for a useful marker to detect it precociously, we aim to evaluate FLCs’ seminal levels in VAR patients in comparison to control subjects (healthy and idiopathic oligoastenozoospermic patients), and to eventually correlate them with CoQ10 and total antioxidant capacity (TAC) seminal levels.

## 2. Results

The main seminal parameters (pH, volume, concentration mln/mL, progressive form percentage and normal morphology percentage) have been evaluated in the present study to better characterize the three groups: normozoospermic controls, varicocele patients and idiopathic oligoastenozoospermic patients (Table 1).

As expected, the idiopathic group showed significantly lower sperm concentration versus the controls and the varicocele group, and significantly lower percentage of progressive motile form in comparison to the controls. Additionally, the varicocele group showed a significantly lower percentage of progressive motile form versus the controls. As regards morphology, only the idiopathic group in our cohort presented significantly lower typical form compared to controls.

**Figure 1:**
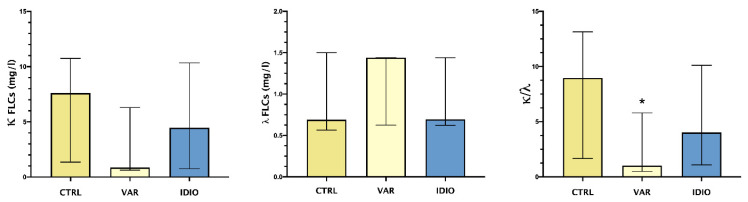
FLCs in the studied groups: median and IQ ranges of seminal plasma levels of κ and λ FLCs and their ratio. Controls seminal fluids (CTRL), varicoceles (VAR), idiopathic oligo- asthenozoospermia (IDIO). * *p* < 0.05.

Figure 1 shows the median and range of seminal plasma levels of κ and λ FLCs and their ratio.

λ FLCs showed a trend toward higher levels in the varicocele group vs. the controls and the idiopathic group. On the other hand, the varicocele patients showed a trend toward lower levels of κ FLCs compared to both the controls and the idiopathic group. This differential pattern of the two types of FLCs reached statistical significance when comparing κ/λ ratio, with significantly lower levels in varicocele patients.

**Figure 2:**
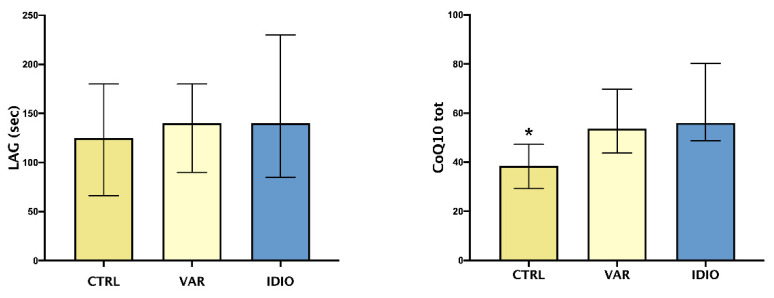
Oxidative stress and seminal fluids: median and IQ ranges of seminal plasma total antioxidant capacity (TAC), expressed as LAG phase, and total coenzyme Q10 (CoQ10 tot). Controls seminal fluids (CTRL), varicoceles (VAR), idiopathic oligo- asthenozoospermia (IDIO). * *p* < 0.05 vs the other two groups.

As shown in Figure 2, TAC, expressed as LAG phase, did not differ between the three groups, whether or not CoQ10 seminal levels were higher in the varicocele and idiopathic groups when compared to the controls.

In varicocele patients, we found significative direct correlations between CoQ10 seminal levels, total sperm count (r^2^ = 0.40, *p* = 0.03) and κ FLCs (r^2^ = 0.46, *p* = 0.03).

No other significant correlations were detected.

## 3. Discussion

The present study follows a previous one [13], which defined FLCs’ detectability in seminal plasma. Our new data confirm the previous findings even on a wider population, with special focus on the binomial relationship between VAR and infertility. While it is common in clinical practice to detect seminal alterations in VAR, the link with male infertility is more elusive. Our study ultimately aimed at shedding some light on this connection, focusing on oxidative stress and inflammation markers, such as FLCs.

VAR is known to be associated with a worse semen quality, due to reduced total sperm count, motility and percentage of normal forms [30]. Pro-inflammatory cytokines IL-18 and IL-37 have been detected in seminal plasma of patients affected by VAR [31]. Inflammatory response, and consequent ROS production, in these patients reduce semen total antioxidant capacity [32]. FLCs are a renowned marker of immune activation [12] and, in a first evaluation in a small cohort of patients with VAR, a reduced κ/λ ratio has been pointed out. We hinted at possible differential patterns in FLC expression between patients with an inflammatory seminal fluid and patients with VAR, the former having a relative prevalence of λ chains compared to the latter; we hypothesized that the low level of κ chains found in the VAR group could be caused by FLCs consumption, or else another signaling system rather than FLCs may be used to summon B cells in semen in VAR.

The present study confirmed many of the findings discussed above: we found that subjects with varicocele had worse sperm cells motility and morphology than controls, we observed a trend in higher λ levels and lower κ levels vs. both controls and the IDIO group. We investigated some parameters connected to oxidative stress and inflammation (i.e., TAC and CoQ10). TAC was evaluated in our previous study, before and after surgical treatment of varicocele [23]. The focus on CoQ10 was due to its anti-inflammatory properties other than the energetic and the antioxidant roles. As anti-inflammatory mechanisms are concerned, it can reduce the production of pro-inflammatory cytokines via inhibition of nuclear factor (NF)-kB gene expression [33] and decrease the modulation of kinases associated with interleukin (IL)-1 receptor and microRNA-146a [34]. It seems that CoQ10 could modulate immune response, via influence on T-cell secretions, as well [34]. Moreover, indirect anti-inflammatory actions can be exerted through the increase in adiponectin secretion [35], in turn related tumor necrosis factor (TNF)-a, IL-6 and c-reactive protein (CRP) decreased secretion [36].

In the present report, TAC did not differ significantly between the three groups, while CoQ10 levels were higher in patients with varicocele and in the IDIO group when compared to controls. TAC assay, in the method employed in this study, estimates only non-proteic and non-enzymatic chain-breaking molecules, as previously reported [37]; the LAG values represent the balance between antioxidant consumption and production, induced by oxidative stress itself. Therefore, it is not surprising that no differences among these groups were discovered, since TAC could be insufficient if considered alone. Anyway, we showed differences in this parameter before and after surgical varicocele repair [28]. Similarly, CoQ10 levels need to be interpreted considering the dual activity of this key molecule, which is involved both in mitochondrial respiratory chain and in oxidoreductive processes, contributing to extra- and intra-cellular balance [38,39]. We found higher levels of CoQ10 in seminal plasma of VAR vs. controls—which is in agreement with previous data—but also in IDIO group. It is hypothesized that the distribution between plasma and cellular compartments was altered in VAR patients; a pathophysiological consequence could be asthenozoospermia, and this mechanism could be shared by IDIO patients. Therefore, CoQ10 could be a useful parameter for its energetic, antioxidant and anti-inflammatory properties. Despite the last aspect justified trials exploring CoQ10 effects on inflammatory markers, in metabolic and cardiovascular diseases [40], the other two roles represented the rationale for some interventional trials, in the field of infertility, which demonstrated a beneficial effect of CoQ10 administration on sperm motility [41,42].

Furthermore, we observed an interesting correlation between both κ chains and CoQ10 levels and total sperm count in the VAR group. Despite not being directly related, κ levels and CoQ10 levels both behaved in the same way when analyzed together with total sperm count, which may foreshadow a pathogenetic link between oxidative stress, inflammation, and infertility in patients with VAR.

Certainly, human infertility appears to be on the rise: in the United States, the National Survey of Family Growth in 2013 found that 9.4% of male Americans aged between 15 and 44 were infertile [43]. Moreover, a trend towards a worse quality of semen has been found dating from the 1970s onwards [44], the most notable features of which were the reduction in total sperm count and seminal fluid volume. Idiopathic infertility still accounts for approximately 30% of the cases [45]. In idiopathic cases, oxidative stress seems to play an important role, and Agarwal et al. supported the idea that it may even be a standalone cause for infertility, coining the new definition Male Oxidative Stress Infertility (MOSI) for such cases [22]. It is then important to assess both oxidative stress and inflammation in seminal fluid when conducting a semen analysis. Regarding oxidative stress, Agarwal et al. suggested the use of the male infertility oxidative stress system (MiOXSYS), which is simple and affordable to use [46], and such criteria should also be pursued when selecting a method to assess the inflammatory status. At present, efforts in this field have been mostly directed towards the detection and measuring of cytokine levels in seminal plasma [31,47,48,49], but results have not been conclusive, as none of the studies in this regard have found a correlation between cytokine levels and seminal parameters. Thus, our findings may be of some importance, as test results are available with shorter test turnaround times than the cytokine assays that are usually analyzed with microtiter plate-based assays, which are highly available, but difficult to collect.

Despite all precautions, the present study may be still subject to certain biases and potential restrictions should be considered. The number of subjects in the three groups is slightly small, so the statistical power of the study is limited; considering the small number of participants, adjusting for possible confounders (such as age) was not feasible. Therefore, our findings will need to be confirmed in a larger population to better understand the role of FLCs in male fertility. Therefore, the study design and the power analysis cannot draw a cause-effect relationship. Moreover, despite the exclusion of possible pathological conditions associated with FLCs’ elevated serum and tissue levels, we did not perform serum evaluation of FLCs. Finally, body fluids FLCs analysis presents a lack of normal value standard reference, as well as a possible interference with data interpretation by product of cellular and protein synthesis in the fluid.

In conclusion, despite several studies on antioxidants and seminal fluid parameters, some new aspects should be clarified to better adapt trials on pathophysiological evidence, since most studies in this field are considered of low quality [50]. In the present study, a peculiar FLC pattern in varicocele patients’ seminal plasma has been detected. Moreover, a correlation between κ chain and CoQ10 has been shown. Our data support a possible role of FLCs as biomarker of inflammation, in turn related to oxidative stress, with the final task to personalize therapy on seminal evaluation.

## 4. Materials and Methods

Patients involved in the study were received in the “Policlinico Universitario A. Gemelli” in an andrological outpatients setting for pregnancy search and/or fertility evaluation and then enrolled after the explanation of research purposes and signature of written consent, according to the Declaration of Helsinki, as revised in 2013. The study protocol was approved by the Institutional Board of our hospital. Adult males aged 18–50 years were included in the study. Among them, 34% were smokers and 66% were non-smokers. Patients’ medical histories and medications have been collected. General, physical, and genital examination were collected in all patients; laboratory exams were also evaluated.

Exclusion criteria were as follows: presence of autoimmune diseases (systemic erythematous lupus, rheumatoid arthritis, multiple sclerosis) or immunomodulatory and immunosuppressive therapy administration; gammopathies of uncertain significance and multiple myeloma; chronic kidney diseases and renal insufficiency; active malignant neoplasms; aspermia; azoospermia; genito-urinary inflammation diagnosed by clinical evidence of lower urinary tract symptoms or microbiological sperm findings; age under 18 or over 50 years.

A total of 65 patients were selected. Varicocele diagnosis was clinical and confirmed by Doppler technique [24]. After 3–5 days of sexual abstinence, semen samples were collected and placed at 37 °C for liquefaction. Standard semen analysis, according to the 2010 WHO laboratory manual for the examination and processing of human semen, 5th edition, has been performed [25]. The 65 patients were divided in 3 groups, as follows:

A. Controls (normozoospermic) 12 patients, median age and interquartile range 34 (33–41) years, BMI 21 (18–23) kg/m^2^, 33% smokers.
B. Varicocele (VAR), 29 patients, median age and interquartile range 33 (26–37) years, BMI 21 (19–26) kg/m^2^, 40% smokers.
C. Idiopathic oligo-, asteno-, oligoastenozoospermia (IDIO), 24 patients, median age and interquartile range 37 (33.5–40.5) years, BMI 24 (20–27) kg/m^2^, 30% smokers. We used the term “idiopathic” referring to seminal abnormalities of unknown etiologies, according to literature indication [51,52,53,54].

To detect FLCs, the collected specimens was centrifuged at 12,000× *g* for 5 min. The upper layer seminal plasma was collected for the determination of FLCs concentrations on OPTILITE instruments using the latex particle enhanced. The *Freelite* assay was composed of two sensitive and specific immunodiagnostic tests to measure kappa (κ) and lambda (λ) free light chains (The Binding Site Group Ltd., Birmingham, UK) following the manufacturer’s instructions. Seminal plasma samples (marked with anonymous code) were not diluted. FLCs were assayed by means of Turbidimetric method. The assay recognized the widest variety of polyclonal free light chains to allow complete detection and accurate sample measurement consisting of two separate measurements, one to detect free κ and the other to detect free λ. The lowest limit of quantification was 0.1 mg/L. The analysis was performed by an operator “blinded” for the clinical information of the handled sample. 

CoQ10 was assayed by HPLC using a Beckman System Gold, as described by Brugè et al. In brief, 10 uL of benzoquinone was added to 100 μL of seminal liquid in order to oxidize all the ubiquinone. Following, total CoQ10 was extracted by adding 500 μL of 2-propanol and vortexing vigorously. After centrifugation at 10,000 g for 2 min at 4 °C, 200 μL of supernatant were directly injected in the column (Supelco LC18 5 μm, 25 cm × 4.6 mm I.D.). The mobile phase was ethanol-methanol (70:30) with flow rate of 1 mL/min. Total CoQ10 was detected at 275 nm and the levels were expressed as ng/mL. 

TAC was determined by spectrophotometric method, according to Rice-Evans and Miller [26], with some modifications as previously described [27]. The method is based on the antioxidants’ inhibition of the absorbance of the radical action 2,2I-azinobis (3-ethylbenzothiazoline-6 sulphonate) (ABTS°) formed by interaction between ABTS (150 μM) and ferrylmyoglobin radical species, generated by activation of metmyoglobin (2.5 μM) with H_2_O_2_ (75 μM). Plasma aliquots were thawed at room temperature and 10 μL of the samples were tested immediately. The manual procedure was used with only minor modifications, i.e., temperature at 37 °C to be in more physiological conditions and each sample assayed alone to carefully control timing and temperature. The reaction was started directly in cuvette through H_2_O_2_ addition after 1 min equilibration of all other reagents (temperature control by a thermocouple probe, model 1408 K thermocouple, Digitron Instrumentation Ltd., Scunthorpe, UK) and followed for 10 min under continuous stirring, monitoring at 734 nm, typical of the spectroscopically detectable ABTS^+^. The presence of chain-breaking antioxidants induces a lag time (the Lag phase) in the accumulation of ABTS^+^ whose duration is proportional to the antioxidants’ concentration. Antioxidant capacity afforded by chain-breaking antioxidants is expressed as length of Lag phase (LAG, sec). Trolox, a water-soluble tocopherol analogue, was used as a reference standard and assayed in all experiments to control the system. Absorbance was measured with an Agilent 8453 UV/Vis spectrophotometer (Palo Alto, CA, USA) equipped with a cuvette stirring apparatus and a constant temperature cell holder. Measurements of pH were made with a PHM84 Research pH meter (Radiometer, Copenhagen, Denmark); the electrode response was corrected for temperature. Unless otherwise stated, experiments were repeated two to three times; qualitatively similar results were obtained with individual values varying <8%. In the Lag mode, the assay mainly measures non-proteic and non-enzymatic antioxidants that are primarily extracellular chain-breaking antioxidants, such as ascorbate, urate, and glutathione.

The statistical analysis was performed using Prism GraphPad 8, San Diego, CA, USA. The sample size was empirical for this observational study. A non-parametric distribution of all data analyzed was evidenced according to the D’Agostino and Pearson test. The differences between κ and λ FLCs in the four groups were evaluated by Kruskal–Wallis test. The same test was used to compare seminal parameters between the four groups. The post hoc analysis was performed using Dunn’s Test. Linear correlation analysis was used to detect any correlation between FLCs and seminal parameters. When the test confirmed a correlation, the Spearman test was used to find out its power. All reported *p* values were two-sided and a value of *p* < 0.05 was considered statistically significant.

## Figures and Tables

**Table 1:** Median and interquartile range of the main seminal parameters used for evaluation of male infertility according to WHO in the three groups. Vol = volume (mL), C = concentrations of spermatozoa in seminal fluid (×10^6^/mL), Total Count = Vol ∗ C (×10^6^), PR = progressive forms (%), *n* = normal forms (%).

	Vol (mL)	C (×10^6^/mL)	Total Count (×10^6^)	PR (%)	*n* (%)
**Controls**	3 (3–4)	43 (27.4–56.5)	120 (81–212)	39 (34.5–45)	6.4 (5.2–7.9)
**Varicocele**	4 (3–4.4)	43 (17.50–67)	149 (80–216)	20 (9–31) *	5.3 (4.3–5.8)
**Idiopathic**	4 (3–4.25)	22.50 (8–61.3) *°	66 (30–294)	19.50 (7–23.25) *	4.9 (3.4–5.7) *

* *p* < 0.05 versus controls, ° *p* < 0.05 versus varicocele.

## Data Availability

Data are available by authors upon request.
